# Minced cartilage implantation for cartilage regeneration: a survey of current clinical practices

**DOI:** 10.1007/s00402-026-06443-9

**Published:** 2026-08-03

**Authors:** Tayfun Yilmaz, Sebastian Leutheuser, Philipp Niemeyer, Peter Behrendt, Philip Roessler, Justus Gille, Marcus Mumme, Stephan Vogt

**Affiliations:** 1 Department of Orthopedic Surgery and Traumatology, University Medical Center Freiburg, Freiburg, Germany; 2 Department of Orthopedic Surgery and Sports Traumatology, Sana Dreifaltigkeits-Krankenhaus Köln-Braunsfeld GmbH, Köln, Germany; 3 Orthopädische Chirurgie München, Munich, Germany; 4 Department of Orthopaedics and Traumatology, University Medical Center Schleswig-Holstein, Kiel, Germany; 5 Orthopedic Surgery Kiel, Kiel, Germany; 6 Gelenkzentrum Mittelrhein, Koblenz, Germany; 7 Klinik für Allgemeine Orthopädie, Unfallchirurgie und Sportmedizin, Regio Kliniken, Pinneberg, Germany; 8 Department of Biomedicine, University Hospital of Basel, Basel, Switzerland; 9 Sportclinic Zurich, Zurich, Switzerland; 10 Department of Sports Orthopaedics, Hessing Stiftung Augsburg, Augsburg, Germany

## Abstract

**Introduction:**

The objective of this study is to evaluate how minced cartilage implantation (MCI) is currently applied in clinical settings, focusing on the selection criteria, procedural execution, handling of concomitant joint pathologies, and postoperative management.

**Material and method:**

An online survey was conducted among all 4,915 members of the German Society for Arthroscopy and Joint Surgery (AGA). Invitations with a survey link were emailed, and the questionnaire was accessible anonymously from January 10 to February 20, 2024. A total of 927 responses were received and analyzed, resulting in a 19% response rate. Demographic data were described descriptively; survey answers were reported as percentages. The 25-item questionnaire, developed by AGA and the Quality Circle for Cartilage Repair & Joint Preservation (QKG e.V.), included single- and multiple-choice questions on experience with cartilage therapy, minced cartilage procedure application and postoperative care, and the overall assessment of this technique within cartilage repair.

**Results:**

MCI has emerged as one of the three most commonly used cartilage repair techniques in the knee joint. Defect sizes up to 4 cm² represent the largest treated group. The application of the procedure is quite heterogeneous, with some practitioners using a shaver and others manually mincing with a scalpel. Fixation methods also vary, including the use of autologous and allogeneic fibrin as well as collagen membranes. The targeted fragment size is largely consistent across users. Postoperative management is similar, with most recommending partial weight-bearing for six weeks.

**Conclusion:**

Minced cartilage implantation has become an increasingly utilized technique for cartilage repair. Although the current evidence has improved - particularly due to the growing number of case series - there are still notable variations in how the procedure is performed.

## Introduction

Cartilage damage often leads to knee pain, regardless of whether the damage is degenerative or traumatic [10]. If cartilage damage remains untreated, spontaneous healing often cannot occur due to the lack of vascularization, which can lead to osteoarthritis in the knee joint [1, 13]. To address this issue, various cartilage repair techniques are available.

Cartilage repair techniques can be broadly divided into two main groups. One group comprises bone marrow stimulation techniques, the most common of which is microfracture. This method, popularized by Steadman, induces the formation of a stem cell-rich blood clot, which primarily produces fibrous cartilage [13, 21]. Combining this method with a collagen matrix can improve clinical outcomes and has shown good long-term results [12, 22]. 

The other group of cartilage repair techniques consists of cell-based methods. Autologous chondrocyte implantation (ACI) is a frequently used approach within this group.

As a two-step procedure, it is suitable for larger cartilage defects and achieves good long-term clinical outcomes [10, 17, 23]. One-step cell-based procedures are also available, such as the transplantation of allogenic or autologous osteochondral cylinders [5]. 

Minced cartilage implantation (MCI) is also a cell-based, one-step procedure that has been increasingly used in recent years. The procedure leads to good short-term results in smaller defects [3, 6, 9, 20]. Long-term successful outcomes are also reported [19]. Nevertheless, MCI has so far not been a standardized procedure, but rather methodologically very heterogeneous in the case series published to date. Autologous cartilage is employed, which is either manually cut into small pieces or fragmented using a shaver. In some instances, additional biomaterials are applied or autologous adjuvants such as platelet-rich plasma are used [2, 9, 19]. 

Although the increasing number of clinical studies and basic research on this topic has enhanced our understanding of MCI in recent years, many questions regarding its clinical application remain unanswered. Currently, there is no standardization of the MCI procedure, unlike ACI, which is standardized through regulatory processes.

The aim of this study is to examine the current state of clinical application. In particular, the focus is on the issues of indication and the application of MCI. Additionally, it is important to evaluate how concomitant pathologies are managed during the application of this procedure. Another aspect to be assessed is the postoperative care.

## Methods

As part of the present study, an online survey (SurveyMonkey, San Mateo, CA, USA) was conducted among all members of the German Society for Arthroscopy and Joint Surgery (AGA). Invitations to participate were distributed via email, containing a survey link, to a total of 4,915 recipients. The questionnaire was accessible online from 10.01.2024 to 20.02.2024 and could be completed anonymously.

A total of 927 questionnaires were filled out. All 927 questionnaires were included in the analysis. A response rate of 19% was achieved, which is considered satisfactory for a survey-based study.

Demographic data were analyzed using descriptive statistics, while responses to the online survey questions were evaluated as percentages.

A sample size calculation was not required, as the sample size matched the total number of active AGA members, all of whom were invited to participate in the online survey.

The questions were developed in collaboration between the German Society for Arthroscopy and Joint Surgery (AGA) and the Quality Circle for Cartilage Repair & Joint Preservation (QKG, e.V.).

**Fig. 1:**
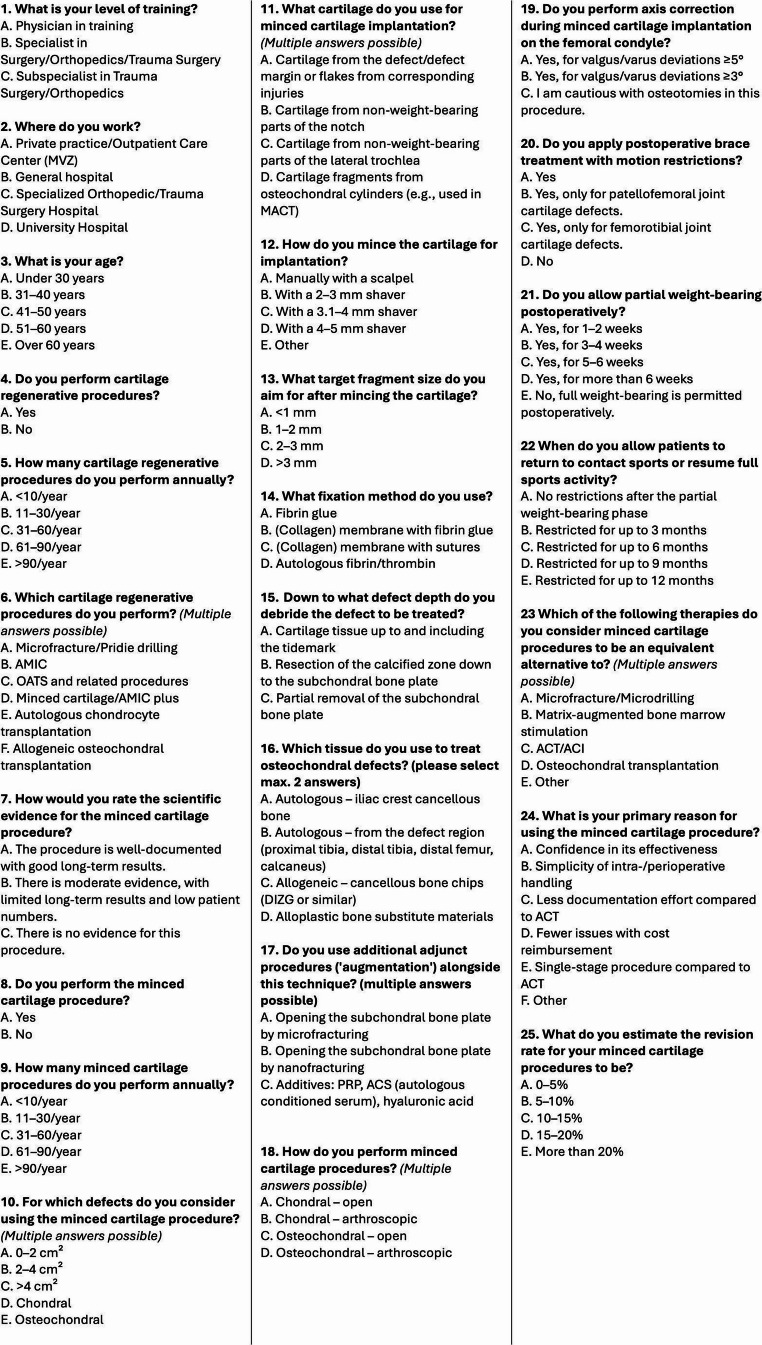
Overview of the questionnaire used in the present survey. The 25-item questionnaire covered participant characteristics, current practice patterns in cartilage repair surgery, indications and surgical techniques for minced cartilage implantation, postoperative management, and surgeons' perspectives on the procedure.

The questionnaire comprises a total of 25 items, including single-choice and multiple-choice questions. It collects data on the level of experience with cartilage therapy, the use and postoperative management of the minced cartilage procedure, as well as the general assessment of this technique within the spectrum of cartilage repair methods Fig. 1.

## Results

The online Survey has a response rate of 19% (927 participants). As the goal of this survey was to evaluate the reality of clinical practice using the minced cartilage implantation (MCI), all participants who did not apply cartilage procedures, particularly those who did not perform MCI, were excluded. A total of 433 participants completed the entire online questionnaire, as these individuals performed MCI.

Among the participants, 13% were residents, and 47% were board-certified specialists in orthopedic surgery and trauma surgery. 40% of the participants were specialized trauma surgeons or orthopedic surgeons. 87% of the participants were between the ages of 30 and 60. Participants had a mean age of 45.6 years (SD = 10.2), with age calculated based on the midpoints of the defined age categories.

A substantial proportion of participants (81%; 747 of 927) reported performing cartilage therapy procedures in their clinical practice. A total of 51% (469 of 927) of the initially surveyed 927 participants performed MCI. However, only 8% of this group performed MCI frequently (more than 30 times per year). The largest group, comprising 55%, performed the procedure rarely (fewer than 10 times per year).

Among the participants who performed cartilage regenerative therapy microfracturing was the most commonly performed procedure (*n* = 607). This was followed by MCI (*n* = 469) and AMIC (*n* = 456). Autologous chondrocyte transplantation is the fourth most common procedure. (*n* = 469). This ranking reflects the proportion of surgeons performing each technique, rather than the overall number of procedures performed.

Although MCI is widely applied, 77% of these participants perceived the quality of the supporting evidence only as moderate.

### Clinical application and handling of minced cartilage implantation

The clinicians using MCI among the participants apply this procedure for the treatment of medium and small defects.

**Fig. 2:**
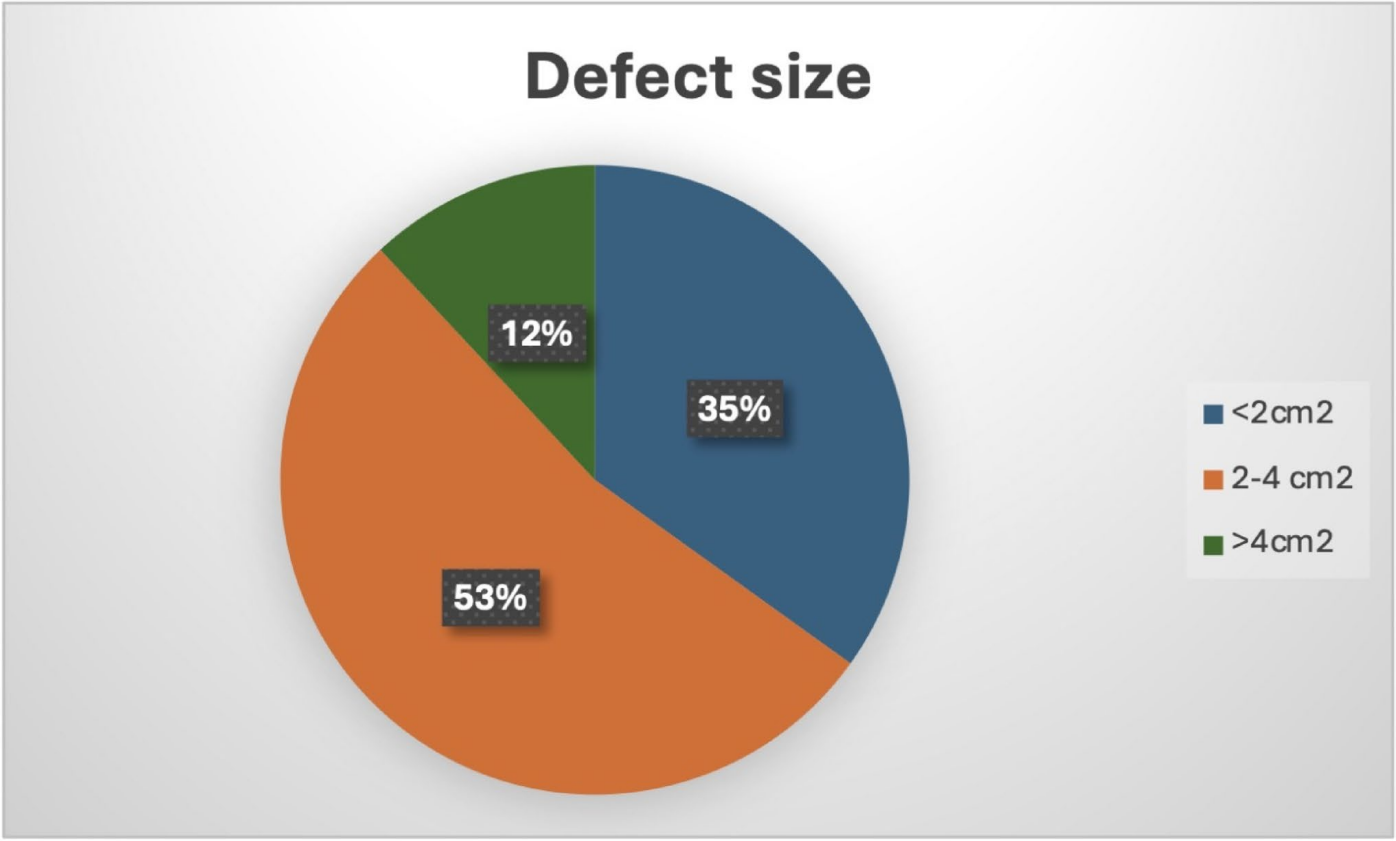
Frequency of treated defect sizes in the context of the MCI procedure

The group with defects ranging from 2 to 4 cm² represents the largest proportion at 53%, followed by defects measuring less than 2 cm² at 35%. A small proportion of 12% of the cartilage defects treated with MCI are larger than 4 cm². [Fig. 2]

In the majority of cases (65%), cartilage is harvested from the defect area or the cartilage flake is processed. In 32% of cases, the cartilage is taken from the non-weight-bearing cartilage portion of the notch or the non-weight-bearing area of the trochlea.

The mincing procedure is performed manually in 36% of the cases. Cartilage fragmentation using a shaver device is applied in 64% of the cases.

However, the present study shows that the size of the shaver used is variable. In 46% of the cases, a shaver with a size of 2–3 mm is used, in 45% a shaver with a size of 3.1–4 mm, and in 9% of the cases, a shaver with a size of 4.1–5 mm.

The question raised in the context of the study regarding the target size of the produced cartilage chips revealed that the group of fragments smaller than 2 mm accounts for the largest proportion at 92.3%. Within this group, 61% aim for a fragment size between 1 and 2 mm. A fragment size of 2 to 3 mm is planned by 7.2% of the participants. Only 0.5% define the required fragment size as larger than 3 mm.

The procedure is performed using an arthroscopic approach in 42% of cases. During defect debridement, 52% of participants debride the calcified layer.

The fixation of the minced cartilage fragments is performed using a membrane with fibrin glue or suture fixation in 30% of cases. Allogenic fibrin glue alone is used in 30%, and autologous fibrin/thrombin is used in 40%.

If there is a bony pathology and bone replacement is necessary, 77.8% use autologous bone, harvested from the iliac crest, femur, or tibia. In 20% of cases, allogenic bone is used. Alloplastic materials are used in 2.2% of cases.

In the treatment group of patients with a concomitant leg axis deviation is treated in 47% of cases when the deviation exceeds 5 degrees. Only 29% perform an osteotomy of the femur or tibia when the leg axis deviation exceeds 3 degrees. Among the participants, 24% are cautious about performing osteotomies in combination with MCI.

### Postoperative treatment of patients undergoing minced cartilage implantation

Following MCI, the majority of patients receive treatment with an orthosis and movement restrictions, with a proportion of 86%.

Partial weight-bearing is recommended in 99% of patients. The recommended duration of partial weight-bearing varies among groups. A duration of 1–2 weeks is recommended by 4% of respondents, while 13% advise 3–4 weeks. The majority, 76%, recommend 5–6 weeks, and 6% suggest a duration of more than 6 weeks.

The management of the permission for return to sports is heterogeneous. A return after three months is recommended by 10%, while 44% suggest six months. Additionally, 27% recommend a return after nine months, and 18% advise waiting until twelve months.

Only 46% of users reported being convinced of the procedure’s effectiveness as a reason for application, while 86% cited the one-step nature of MCI compared to ACI as a primary reason for its use. Overall, the participants consider MCI a viable alternative to ACI, AMIC, and microfracturing.

## Discussion

The survey highlights the heterogeneous application of minced cartilage implantation (MCI) and reflects the limited and inconsistent evidence base. While many clinicians report using MCI, around one fifth apply it regularly in their practice. The procedure is most often employed for small to medium-sized defects, yet the techniques of cartilage harvesting, fragmentation, and fixation vary considerably. Despite ongoing uncertainties regarding its effectiveness, MCI is frequently favored because of its one-step nature compared with more complex alternatives.

Microfracture remains the predominant treatment method for cartilage defects, which is consistent with an evaluation of the German Cartilage Registry. In comparison with the evaluation of the cartilage registry from 2016, the emergence of the MCI procedure indicates a shift in the frequency of use among cartilage regenerative therapies. This observation is noteworthy, as the evidence supporting these approaches remains limited.

There are currently very few comparative studies that help to position the MCI technique in relation to established cartilage regenerative methods [2, 24]. A recent matched-patient analysis demonstrated that autologous matrix-induced chondrogenesis (AMIC) yields superior mid-term outcomes compared to hand-minced autologous cartilage implantation (MCI) fixed with fibrin glue for the treatment of knee chondral defects, with significantly better pain relief, functional scores, and quality of life measures [2]. 

In addition there remains a lack of evidence regarding the surgical techniques of MCI. These techniques can be broadly categorized into two main groups: membrane fixation and fibrin fixation. At this time point, no studies have compared these two procedures. Recent studies reporting on the clinical outcomes of MCI have included both techniques within their publications without performing subgroup analyses, reflecting the heterogeneity of the procedure in the current literature [14, 19]. Overall, the results of MCI in smaller cartilage defects have been promising [9]. Therefore, the German Cartilage Repair Workgroup recommended MCI as a potential treatment for cartilage defects up to a size of 4 cm² [18]. 

It is known that extracellular matrix production improves when the cartilage fragment size is smaller [4]. This aspect is considered, as the respondents in our study also prefer the smallest possible fragment size, below 2 mm. Even though a fragment size of less than 2 mm is intended, variable and larger sizes are sometimes used during extraction and fragmentation with the shaver.

The optimal number of viable, intact chondrocytes necessary to ensure successful outgrowth of transplanted cartilage tissue remains unclear due to a lack of conclusive evidence. Current evidence suggests that cartilage mincing does not significantly affect cellular migration, tissue outgrowth, or extracellular matrix synthesis when compared to cartilage harvested without mincing [7]. Chondrocytes harvested and minced with an arthroscopic shaver have been shown to remain viable and retain their proliferative capacity [11]. However, in bovine cartilage, the mincing procedure using a shaver reduces chondrocyte viability compared to manual mincing [15]. A comparison of these two mincing methods on porcine cartilage tissue revealed a similar effect: arthroscopic mincing results in reduced chondrocyte viability and diminished quality of the cartilaginous repair tissue [8]. The conclusion of these studies appears to be that mincing with a shaver does not significantly affect proliferative capacity but does reduce cell viability and the quality of the regenerated tissue. Overall, it remains unclear to what extent findings from in vitro studies can be translated into concrete guidance for clinical application and outcomes, which is why both techniques continue to be used in practice.

There are currently no standardized postoperative rehabilitation protocols for patients undergoing minced cartilage implantation in the knee joint. Nevertheless, most clinicians follow a similar approach, typically recommending partial weight-bearing for a duration of six weeks.

A limitation of this survey is the potential for selection bias, as participants are often those already engaged in academic or clinical work on cartilage. Another limitation is that the survey did not differentiate between tibiofemoral, trochlear, and retropatellar cartilage defects in the context of MCI application. Previous studies have shown that methodological differences exist depending on the defect location, affecting how MCI is performed. Future research should report the specific techniques in detail and ideally provide prospective, comparative data to establish more robust evidence.

## Conclusion

Minced cartilage implantation has become an increasingly utilized technique for cartilage repair. Although the current evidence has improved - particularly due to the growing number of case series [16] - there are still notable variations in how the procedure is performed. Further studies are necessary to strengthen the evidence for the individual components of the technique in order to enable its standardization in the future.

## Data Availability

The data collected and analyzed in this study are available from the corresponding author upon reasonable request.
